# Molecular Survey and Genetic Diversity of Hemoplasmas in Rodents from Chile

**DOI:** 10.3390/microorganisms8101493

**Published:** 2020-09-29

**Authors:** Amir Salvador Alabí, Gustavo Monti, Carola Otth, Paulina Sepulveda-García, Melissa Sánchez-Hidalgo, Victória Valente Califre de Mello, Rosangela Zacarias Machado, Marcos Rogério André, Pedro Bittencourt, Ananda Müller

**Affiliations:** 1 Instituto de Ciencias Clinicas Veterinarias, Facultad de Ciencias Veterinarias, Universidad Austral de Chile, Valdivia 4090000, Chile; amir_cordova@hotmail.com (A.S.A.); paulina.sepulveda.garcia@gmail.com (P.S.-G.); 2 Insituto de Medicina Preventiva Veterinaria, Facultad de Ciencias Veterinarias, Universidad Austral de Chile, Valdivia 4090000, Chile; gustavomonti@uach.cl; 3 Instituto de Microbiologia Clinica, Facultad de Medicina, Universidad Austral de Chile, Valdivia 4090000, Chile; cotth@uach.cl; 4 Instituto de Ciencia Animal, Facultad de de Ciencias Veterinarias, Universidad Austral de Chile, Valdivia 4090000, Chile; melisanchez_rh@hotmail.com; 5 Departamento de Patologia, UNESP, Teriogenologia e Saúde Única—Faculdade de Ciências Agrárias e Veterinarias Universidade Estadual Paulista (FCAV), Jaboticabal, São Paulo 14884-900, Brazil; vick_vvc@hotmail.com (V.V.C.d.M.); rzacariasmachado@gmail.com (R.Z.M.); mr.andre@unesp.br (M.R.A.); 6 One Health Center for Zoonoses and Tropical Veterinary Medicine, Department of Biomedical Sciences, Ross University School of Veterinary Medicine, Basseterre, Saint Kitts and Nevis; pbittencourt@rossvet.edu.kn

**Keywords:** hemotrophic mycoplasmas, rodentia, 16S rRNA, South America

## Abstract

Even though hemotrophic mycoplasma (hemoplasma) infections are well documented in a wide variety of hosts worldwide, there is a gap in the knowledge aobut hemoplasmas in rodents. This study aimed to molecularly survey and investigate the genetic diversity of hemoplasmas in rodents from Chile. Synanthropic and wild rodents (*n* = 74) were captured in the southern province of Valdivia (Corral, Valdivia, Riñihue, and Reumén localities). Spleen samples were submitted to a conventional PCR for hemotrophic *Mycoplasma* spp. targeting the 16S rRNA gene (800 bp), followed by sequencing, phylogenetic, and genetic diversity analyses. The overall occurrence of hemotrophic mycoplasmas in rodents from Valdivia was 24.5% (18/74) [95% CI (14.5; 34.1)]. Hemoplasmas were detected in *Mus musculus* (1/4), *Rattus norvegicus* (1/16), *Abrothrix longipilis* (7/13), *A. olivaceo* (6/8), and *Oligoryzomys longicaudatus* (3/10). The nucleotide polymorphism analysis of the targeted 16S rRNA region showed low diversity, with two genotypes and a high identity to the variants detected in wild rodents from Brazil. Hemoplasmas are described for the first time in rodents from Chile with a moderate occurrence and low 16S rDNA genetic diversity within the sampled rodent population. The detected hemoplasma genotypes were specific to rodents and were not shared with other mammals.

## 1. Introduction

Hemotrophic mycoplasmas, also known as hemoplasmas, are Gram-negative, uncultivable pleomorphic bacteria that lack a cell wall and belong to the genus *Mycoplasma*, Mollicutes Class [1,2] Hemoplasmas are cocci that attach to the surface of red blood cells [3,4]. Their pathogenicity can range, depending on the hemoplasma and mammalian host species, from acutely life-threatening haemolytic anemia to chronic infection with no apparent clinical manifestation [5,6,7,8]. In the last decade, several new hemoplasma genotypes were described in wild animals worldwide [9,10,11,12,13,14,15,16,17,18,19,20,21].

Rodentia is the most diversified and widespread order of mammals. Rodents act as hosts for a variety of zoonotic pathogens [22] and several species of ticks and fleas [23,24,25,26]. Hemotrophic mycoplasmas in rodents have been reported in Brazil [14,15,27,28,29,30], Hungary [22], Japan [2,20,31], and Israel [32]. While *Mycoplasma coccoides* and *M. haemomuris* are the most commonly recognized hemoplasmas in the blood of wild and laboratory rodents, respectively [33,34,35,36], novel *Mycoplasma* spp., which has yet to to be fully characterized, have been described in rodents from Brazil [14,15,27,28] and Japan [20]. These agents rely on the persistence of low bacterial loads for long-term coexistence with their rodent hosts [32]. Even though most hemotrophic mycoplasmas are host specific, interspecies infections [37] and species with zoonotic potential are reported in rodents [38,39,40], supporting the importance of studying hemoplasmas in those hosts.

In Chile, hemoplasmas have been reported in dogs [41,42,43], cats [43,44,45,46], camelids [47], and associated fleas [45]. In wild animals, even though hemoplasmas were detected in Darwin’s foxes (*Lycalopex fulvipes*) [48], bats (*Histiotus macrotus*, *Histiotus montanus* and *Myotis chiloensis*) [49], and kodkods (*Leopardus guigna*) [46], there are no studies on rodents from Chile so far.

This study aimed to molecularly survey and investigate the genetic diversity of hemoplasmas in rodents from Chile.

## 2. Materials and Methods

### 2.1. Study Site

**Figure 1:**
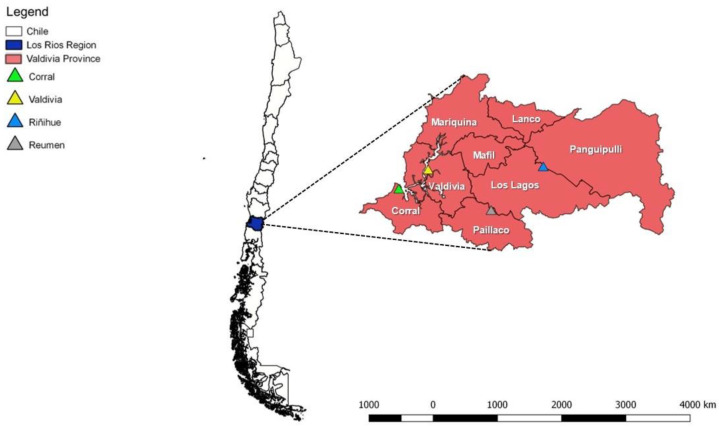
Dairy farms where rodent sampling was performed, Region de Los Rios, Valdivia, Chile.

This study was approved by the Universidad Austral de Chile (UACh) Bioethics Committee (Uach/1141070) in July 2014. Animals were sampled by convenience from dairy farms. The sampling was performed within the Valdivia Province, southern Chile, and included the localities of Corral (Arica Interior [39°53′18.806″ S 73°26′25.272″ W] and Huiro [39°57′52.0″ 73°38′55.3″]), Valdivia city (39°47′21.48″ S 73°14′37.32″ W), Reumén (39°59′54.96″ S 72°49′18.12″ W), and Riñihue (39°46′25.32″ S 72°28′20.28″ W) (Figure 1).

### 2.2. Rodent Trapping and Sampling

Between August 2014 and December 2016, traps (20 cm~20 cm~60 cm Tomahawk cages) were placed in areas with indicators of rodent activity, within the farms (in areas where cattle feed and dwell) as well as in forests. Traps were positioned at distances of 50 to 150 m, with at least 5 m between cages.

The bait consisted of oatmeal and vanilla flavoring and was placed in the traps which were then set in the afternoon and checked every morning for 4 days. All trapped animals were transported, euthanized, and processed in the Instituto de Patología Animal, Facultad de Ciencias Veterinarias, Universidad Austral de Chile. The capture, management, and euthanasia of rodents was conducted in compliance with the specifications of the American Society of Mammologists.

Euthanasia was performed by administrating inhaled Isoflurane, followed by an intraperitoneal injection of a combination of Xylazine and Ketamine, using a single lethal dose [50]. The rodents were identified by their morphological characteristics, gender, maturity, weight, and several measurements (head-body, tail, and ear pinnae) [51]. The spleen of each rodent was immediately removed aseptically after euthanasia. Spleen samples were preserved in 70% alcohol (Merck©, Kenilworth, NJ, USA) and stored at −20 °C, until molecular analyses.

### 2.3. DNA Extraction from Rodent Spleen Tissues

The spleens were thawed at room temperature and diced in portions of 15 mg. Each spleen fragment was covered in aluminum foil and submerged in liquid nitrogen, then powdered in a mortar. DNA extraction was performed with the “Tissue DNA Kit” (E.Z.N.A. Omega BioTek^®^, Norcross, GA, USA), according to the manufacturer’s instructions, to obtain 100 µL of purified DNA. DNA concentration and absorbance ratio (260/280 nm) were determined using a spectrophotometer (NanoDrop ND-1000 Thermo Scientific©, Waltham, MA, USA). For every 20 extractions, nuclease-free water (Thermo Scientific©) was used as a template to verify cross-contamination. The DNA was stored at −20 °C before performing the PCR assays.

### 2.4. Endogenous PCR for Mammals

A mammal *irbp* (interphotoreceptor retinoid-binding protein) endogenous gene-based PCR was used to check the integrity of the DNA template [52]. The reaction mixture was composed of 5 µL Gotaq^®^ Green Master Mix 2X (Promega^®^, Madisson, WI, USA), 10 nM of each primer (IRBP-F and IRBP-R), and 2 µL of template DNA brought to a total volume of 10 µL with nuclease-free water (Thermo Scientific©). Primers and the thermic protocol are described in Table 1.

### 2.5. Molecular Detection of Hemotrophic Mycoplasma *spp.* 16S rDNA

Positive samples for the *irbp* gene were submitted to a previously described [53] conventional (c) PCR protocol aiming to amplify 800 bp of the hemotrophic *Mycoplasma* spp. 16S rRNA gene. All PCRs were performed with nuclease-free water (Thermo Scientific©, Waltham, MA, USA) as a negative control. *Mycoplasma wenyonii* and ‘Candidatus *Mycoplasma haemobos*’ DNA samples from naturally infected cattle [54] were used as positive controls. The reaction mixture for the PCR Protocol was composed of 1.25 U of Taq DNA Polymerase (Thermo Scientific©, Waltham, MA, USA), 0.2 mM of each deoxynucleotide, 1.5 mM of MgCl_2_, 0.5 µM of each primer, and 5 µL of template DNA brought to a total volume of 25 µL with nuclease-free water (Thermo Scientific©, Waltham, MA, USA). The thermic protocol is described in Table 1.

Conventional PCR results were visualized in 1% agarose gel stained by Ethidium bromide ultrapure solution (Life Technologies©, Carlsbad, CA, USA). 16S rDNA amplicons that presented a good band quality in the agarose gel were purified using a Silica Bead DNA Gel Extraction Kit (Fermentas, São Paulo-SP, Brazil), following the manufacturer’s instructions, and sent to the Center of Biological Resources and Genomic Biology (CREBIO, Jaboticabal, SP, Brazil) for sequencing by Sanger’s method with ABI PRISM 3700 DNA Analyzer (Applied Biosystems©, Foster city, CA, USA).

### 2.6. BLAST Analysis

Electropherograms were submitted to PhredPhrap analysis to determine nucleotide composition [55], with Phred quality scores (peaks around each base call) established as higher as 20 (99% accuracy of the base call). The percentage of identities were obtained using nBLAST [56]. The similarity of the present study’s sequences with those previously submitted in GenBank was determined by percentage identity and E-value, and only the best hits were used. The sequences were submitted to GenBank [57] under accession numbers MT345318-MT345325.

### 2.7. Phylogenetic Analysis

For phylogenetic analysis, the Bayesian model was inferred with MrBayes (3.2.2) on XSEDE [58] via CIPRES Web portal [59]. The best evolutionary model was selected with jModelTest2 (version 2.1.6) on 11 XSEDE [60], under the Akaike Information Criterion (AIC) [61]. The Bayesian analysis was made with 10^6^ generations and several substitutions and the posterior probabilities with 10,000 repetitions, chains = 4, Number of chains per microprocessor = 1, Burn-in = 25%, and an average standard deviation of split: less than 0.01. The phylogenetic tree was edited with Treegraph (2.0.56-381 beta) [62] and cluster highlighting was created with BioRender.com [63].

### 2.8. Genotype Analysis

For nucleotide polymorphism (genotype) analysis, eight 16S rDNA hemotrophic *Mycoplasma* spp. sequences obtained from this study were evaluated comparatively to 29 hemoplasma sequences previously detected in rodents around the world (Table S1). Polymorphism nucleotide analysis of 16S rRNA sequences was performed using DnaSP v5 [64]. Genotype diversity (Gd), number of genotypes (n), and nucleotide diversity (Pi) were obtained to explore genetic variation among the hemoplasma sequences of the sampled rodents. The genotype network was generated with PopArt [65] with TCS network.

### 2.9. Splits Network Analysis

Splits network analysis was performed with the Neighbor-net method and uncorrected P distance with SplitsTree v4.11.3 [66], using eight 16S rDNA hemoplasma sequences obtained from rodents in this study and 22 rodent associated hemoplasma sequences from other localities around the world and retrieved from BLAST to create a genotype network. All sequences were previously aligned in BioEdit v. 7.0.5.3 (Carlsbad, CA, USA) [67] and only the ones with an optimal alignment with other sequences were used. Sequences KT215641 and KT215642 were not used for the Splits network.

### 2.10. Data Analysis

To determine the occurrence of hemoplasmas in rodents from Valdivia, Chile, PCR-positive rodents were divided by the total number of animals and multiplied by 100. Occurrence was also determined per locality. The observed frequencies were expressed in percentages and the 95% Confidence Interval (CI) was calculated accordingly to a previously described equation [68].

## 3. Results

### 3.1. Rodents, Amplifiable DNA, and Hemoplasma Survey

Among the 74 sampled rodents, 35.2% (26/74) were trapped in Corral, 8.1% (6/74) in Valdivia, 23% (17/74) in Reumén, and 33.8% (25/74) in Riñihue localities. Four rodent genera were identified: 4.1% (4/74) were represented by the house mouse (*Mus musculus*), 17.6% (16/74) by the brown rat (*Rattus norvegicus*), 18.9% (18/74) by the black rat (*Rattus rattus*), 16.2% (13/74) by the long-haired grass mouse or long-haired akodont (*Abrothrix longipilis*), 10.8% (8/74) by the olive grass mouse (*A. olivaceus*), 12.2% (10/74) by the long-tailed mouse (*Oligoryzomys longicaudatus*), and 2.7% (5/74) were not identified on a species level, being identified as akodont (*Abrothrix* spp.). All 74 DNA samples tested positive for the *irbp* mammalian endogenous gene (Mean and Standard Deviation (SD) with a DNA concentration = 159.60 ng/µL ± 212.09 ng/µL; mean and SD 260/280 ratio = 2.12 ± 0.21).

An overall proportion of 24.5% (18/74) [95% CI (14.5–34.1%)] of the rodents were positive for hemoplasmas based on PCR assays targeting 16S rRNA gene. Positive rodents were found within the four geographic sampled areas: Corral = 1.4% (1/26) [95% CI (0–4%)]; Valdivia 2.7% (2/6) [CI 95% (0–6.4%)]; Reumén 4.1% (3/17) [CI 95% (0–8.5%)]; Riñihue 16.2% (12/25) [CI 95% (7.8–24.6%)].

While 2.8% (2/74) [95% CI (0-6.4%)] of the hemoplasma-positive samples were from synanthropic rodents, 21.7% (16/74) [95% CI (12.24-31%)] of hemoplasma-positive samples were detected within wild rodents. Hemotrophic *Mycoplasma* spp. were detected in the majority of the rodent species captured in this study, with the exception of *R. rattus* and *Abrothrix* sp. The occurrence of hemoplasmas in each rodent species was the following: *Mus musculus* 1.8% (1/4) [95% CI (0–67.43%)]; *Rattus norvegicus* 6.25% (1/16) [95% CI (0–18.11%)] and *Rattus* 0% (0/18) [95% CI (0%)], resulting in an overall occurrence of 7.65% (2/38) [95% CI (0–12.36%)] among synanthropic rodents. Regarding wild rodents, the occurrence rates were as follows: *Abrothrix longipilis* 53.84% (7/13) [95% CI (25.13–80.78%)]; *Abrothrix olivaceo* 75% (6/8) [95% CI (44.99–100%)]; *Abrothrix* sp. 0% (0/5) [95% CI (0%)]; *Oligoryzomys longicaudatus* 30% (3/10) [95% CI (1.6–58.4%)], resulting in an overall occurrence of 44% (16/36) [95% CI (28.21–60.68%)] among wild rodents.

### 3.2. BLAST Analysis

Eight amplicons obtained by the hemoplasma 16S rRNA-PCR protocol were sequenced. Six sequences (MT345318, MT345320, MT345322, MT345323, MT345324, MT345325) presented 98–99% identity with hemotrophic *Mycoplasma* spp. detected in *Delomys dorsalis* rodents from Brazil (KT215622) [15], and two sequences (MT345319, MT345321) presented 98–99% identity with hemotrophic *Mycoplasma* spp. detected in *Necromys lasiurus* rodents from Brazil (KT215623) [15]. The sequences were compared on BLAST on the 04/13/2020: only the best hits were used for percentage of identity, and an E-value of 0 was obtained for all compared sequences.

### 3.3. Phylogenetic Analysis

**Figure 2:**
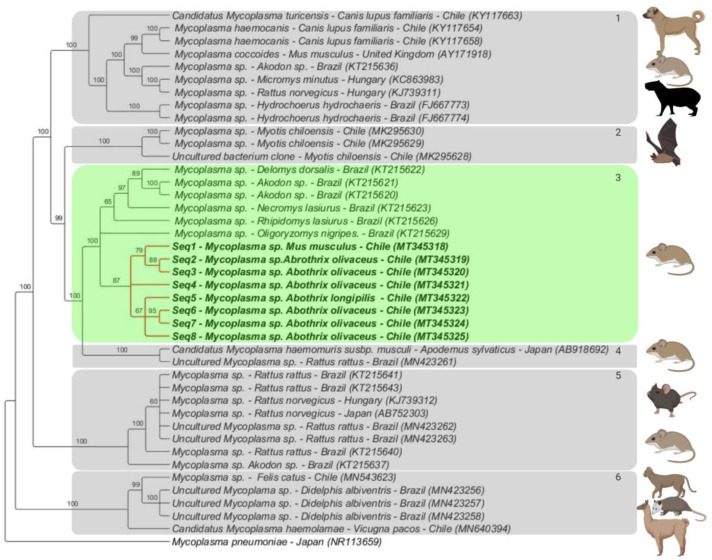
Phylogenetic tree based on an alignment of sequences of hemotrophic *Mycoplasma* spp. 16S rDNA (800 bp) sequences, using the Bayesian method and the TIM3+I+G evolutionary model. Numbers at nodes correspond to posterior probability values. Sequences detected in the present study are bolded. *Mycoplasma pneumoniae* was used as an outgroup.

The Bayesian phylogenetic inference supported the formation of six *Mycoplasma* clusters (Figure 2). Cluster #1 was comprised by ‘Candidatus *Mycoplasma turicensis*’ obtained from domestic dogs from Valdivia, Chile (KY117663, KY117654 and KY117658) along with European (AY171918, KC863983, KJ739311) and Brazilian (KT215636, KC863983, FJ667773 and FJ667774) rodents; cluster #2 encompassed sequences detected in bats from Chile (MK295630, MK295629 and MK295628); cluster #3 covered sequences detected in wild rodents from Brazil (KT215620, KT215621, KT215622, KT215623, KT215626, and KT215629) and hemoplasma sequences detected in rodents from the present study (MT345318-MT345325); cluster #4 included sequences detected in rodents from Japan (AB918692) and Brazil (MN423261); Cluster #5 contained sequences detected in rodents from Brazil (KT215637, KT215640, KT215641, KT215643, MN423262, and MN423263), Hungary (KJ739312) and Japan (AB752303); cluster #6 included hemoplasmas previously detected in alpacas (*Vicugna pacos*) (MN540394), in a domestic cat (MN543623) from Chile and in opossums from Brazil (*Didelphis albiventris*) (MN423256-MN423258). The average standard deviation of split was less than 0.01 (0.009), all Bayesian trees converged.

### 3.4. Genotype Analysis

**Figure 3:**
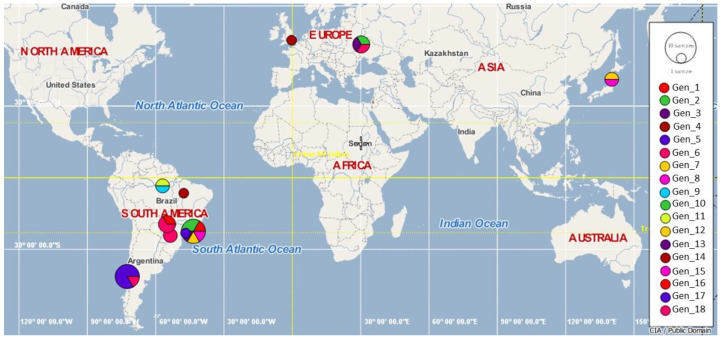
Geographic distribution of hemoplasmas 16S rDNA genotypes in rodents worldwide, including the two genotypes (#17 and #18) found in the present study, in the Valdivia province, Southern Chile.

According to the polymorphism analysis of eight 16S rDNA hemotrophic *Mycoplasma* spp. sequences obtained in this study and 29 hemoplasma sequences previously detected in rodents worldwide, 18 genotypes of *Mycoplasma* spp. were found; while two of them were detected exclusively in southern Chile (#17 [MT345318-MT345320, MT345322- MT345325] and #18 [MT345321]), the remaining were distributed around the world (#1 [KT215622], #2 [KT215623 and KT215621], #3 [KT215620], #4 [KT215626], #5 [KT215629], #6 KT215643, KT215640, KJ739312, MN423262 and MN423263], #7 [KT215636], #8 [KT215637], #9 [FJ667773], #11 [FJ667774], and #16 [MN423261] were described in Brazil; #6 [KT215643, KT215640, KJ739312, MN423262 and MN423263], #10 [KC863983] and #13 [KJ739311] in Hungary; #14 [AY171918] in the United Kingdom; #12 [AB752303] and #15 [AB918692] in Japan (Table 2, Figure 3).

**Figure 4:**
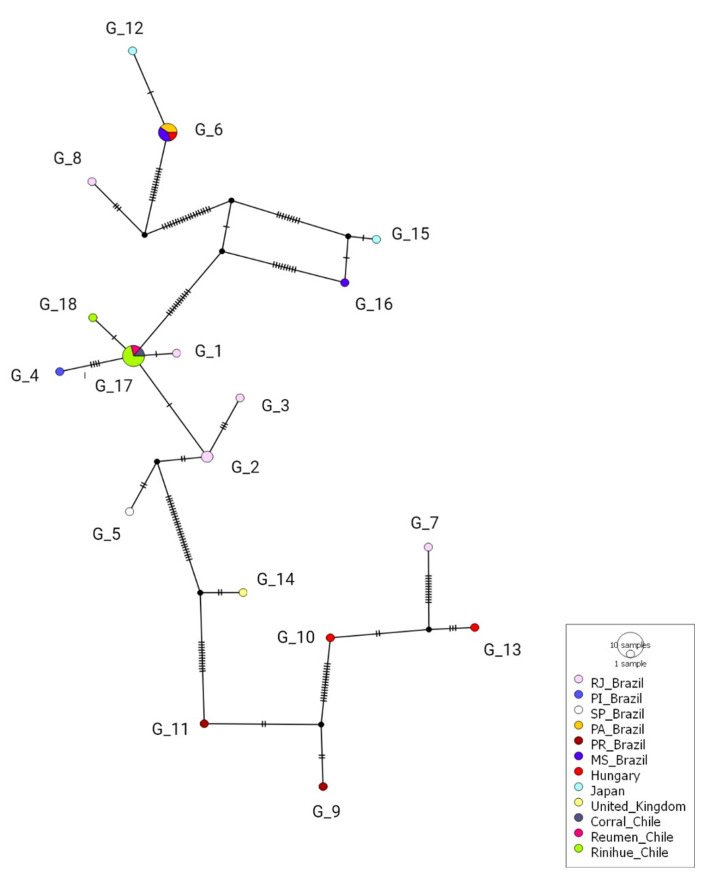
Genotype network for *Mycoplasma* spp. 16S rDNA sequences detected in rodents from the Valdivia province, Chile (genotypes #17 and #18), combined with hemoplasma sequences that were previously detected in Rodentia worldwide. Each small dash represents mutations. Dark circles represent median vectors. The genotype network was generated with DNAsp data followed by analysis in PopArt using geographic coordinates and a TCS network.

Genotypes from Chile were positioned between a common median vector—interpreted as a possible extant unsampled sequence or an extinct ancestral sequence [69]—(differing by several mutational events) and genotype #2 from Rio de Janeiro, Brazil (which differed by one mutation). Genotype #18 in Chile was observed in Riñihue and was derived from genotype #17, also from Chile, obtained from rodents in Corral, Riñihue, and Reumén. Overall, the Brazilian genotypes were highly diverse and widely distributed within the Genotype network (Figure 4).

### 3.5. Splits Network Analysis

**Figure 5:**
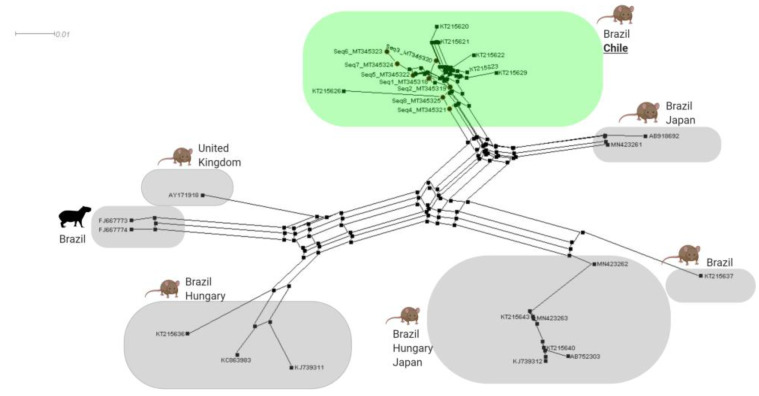
SplitsTree analysis generated by Neighbor-net and uncorrected P distance of hemotrophic *Mycoplasma* spp. 16S rDNA sequences obtained from rodents sampled in the present study, compared to hemoplasmas previously detected in Brazil, Hungary, Japan, and the United Kingdom. Present study Accession numbers (Chile) are preceded by Seq 1–8, respectively, englobed in the green cluster.

According to the Splits network analysis the hemoplasma 16S rDNA sequences detected in the present study were closely related to hemoplasmas detected in wild rodents from Brazil. The sequences from rodents in Chile were related to those detected in specimens of *Akodon* spp. (KT215620; KT215621), *Delomys dorsalis* (KT215622), *Necromys lasiurus* (KT215623), albeit far from those detected in *Rhipidomys macrurus* (KT215626) (Figure 5). The other clusters contained more homogenous groups of hemoplasmas sequences detected in wild or synanthropic rodents, and an exclusive group for the capybaras was observed. There is some evidence of a possible gene transfer and/or recombination within the 16S rRNA from rodents’ hemoplasmas.

## 4. Discussion

Previous studies supported the fact that hemotrophic mycoplasmas have evolved along with a broad diversity of mammal species [3,70]. Chile is not an exception, since hemoplasmas have already been reported in dogs, cats, South American camelids, and wild animals (foxes, kodkod, and bats) [43,45,46,47,48,49,71,72]. To our knowledge, this is the first report of hemotrophic mycoplasmas in rodents from Chile.

While the worldwide rodent hemoplasma prevalence ranges from 11.1% in Japan [20] to 67.30% in Hungary [22], a similar occurrence, from 15% [29] to 63.5%, is observed in South America. The observed molecular occurrence of hemoplasma in rodents from Chile (24.5%) is similar to that previously reported in rodents from Brazil (21.9–25%) [15,28]. The hemoplasma prevalence may be a reflection of various biological factors, such as the group of animals (free-ranging vs. captive animals) [30], gregarious behavior of the hosts [14], age [73], and habitat types (e.g., undisturbed sites vs. disturbed sites) [12].

Herein, a higher occurrence of hemoplasmas was observed in wild rodents (21.7%) compared to synanthropic ones (2.8%). This might be explained by the host specificity of hemoplasma towards wild rodents [28], or it may also be due to their behavior. Wild rodents are more adapted to live far from human facilities, in forests, compared to synanthropic rodents that live in anthropized areas [12,14,74,75]. The highest occurrence of hemoplasma infection in wild rodents could be partially attributed to aggressive interactions among these animals due to lower food availability in forests compared to urban areas—since the main route of rodent-associated hemoplasma transmission seems to be direct through blood and saliva [32]. Furthermore, the higher availability of food for synanthropic rodents might improve their immune response against hemoplasma infection [76].

Even though hemoplasmas were previously detected in ectoparasites [2,23], such as in *Polyplax* spp. lice [2,28,77], *Synosternus cleopatrae* fleas [32], and *Amblyomma* spp. ticks [28], suggesting they might play a role in the transmission among rodents, vector capacity, and competence are yet to be proven. Unfortunately, ectoparasites were not sampled in this study. As mentioned before, there is evidence that rodent-rodent transmission through aggressive interaction is the main route of transmission [32]. Future studies in Chile should explore the routes of hemoplasma transmission among rodents.

In the present study, the 16S rDNA hemoplasma sequences detected in rodents were closely related to those previously detected in rodents from Brazil and Japan. Due to similarities between hemotropic *Mycoplasma* spp. nucleotide sequences obtained in this study and sequences previously detected in other rodents from Brazil [15] (supported by the genotype network and the Splits network analysis, where hemoplasma sequences from both countries were enclosed in a single and homogeneous group), one can infer that both originated from a common ancestor. Alternatively, Chilean hemoplasma genotypes may have derived from those found in rodents from Brazil or vice-versa.

A low number of 16S rDNA hemoplasma genotypes (n = 2) were found among rodents sampled in southern Chile. The rodent-associated hemoplasma genotypes from southern Chile showed a high level of similarity to one another. In contrast, nine genotypes were observed in sequences retrieved from two studies in Brazil [15,28] which was supported by the genotype network from our study, highlighting the high genetic diversity among hemoplasmas in rodents from Brazil. These differences may be due to the number and population structure of the sampled rodents, biomes, environmental conditions, and abundance of ectoparasites. Specifically, the surveys in rodents from Brazil [15,28] covered a much broader geographic area in the country, encompassing various Brazilian states, including but not restricted to Piaui (97,116 mi²), Para (481,700 mi²), Rio de Janeiro (16,871 mi²), Sao Paulo (95,834 mi²), Parana (76,956 mi²), and Mato Grosso do Sul (137,887 mi²)] within different biomes, while Chilean samples were all collected within the Valdivia province (3,937mi²). A wider geographic sampling and a broader molecular characterization including the full 16S rRNA gene and the more evolving genic regions (e.g., 23S [78], RNAseP [53], ITS [79]) are required to further characterize Chilean rodent-associated hemoplasma genotypes [20].

In the Splits network graphic, which was intended to compare the distance between the sequences by the chosen parameters (Neighbor-net and uncorrected P distance), some evidence of transfer and/or recombination of the 16S rRNA gene was noticed, according to Huson (2006) [80]. Even though there are adjacent dots within the Chilean and Brazilian sequences, potentially related to gene recombination, a reticulate network [80] would be recommended to confirm this observation. Indeed, 16S rRNA gene transfer was reported by Deshuillers (2014) [81] in an ovine hemotrophic *Mycoplasma* in which two different copies of the gene were found within its genome. It is possible that a 16S rRNA transfer or recombination occurs for hemoplasmas and future studies—including the whole 16S rRNA gene and other less conserved ones—should be performed for evaluating the recombination.

Phylogenetic analysis demonstrated that all hemotrophic *Mycoplasma* spp. sequences obtained from rodents in the Valdivia province were grouped together (cluster #3). The majority of the obtained hemoplasma sequences were detected in wild rodents; the only *M. musculus*-associated sequence was closely positioned to Chilean wild rodent hemoplasma sequences. In contrast, previous studies in Brazil [15,28] reported that hemotrophic *Mycoplasma* infecting wild rodents is restricted to this group and does not seem to infect *R. rattus* or *M. musculus*. Future investigations should include a larger number of rodents to evaluate if the genotype patterns between wild and synanthropic groups in Chile are different from those described in Brazil.

Although the present work did not investigate the occurrence and molecular identity of hemoplasmas in sympatric domestic and wild mammals in areas where the rodents were sampled, there was no molecular evidence of cross-infection or sharing of genotypes among the sampled rodent-associated hemoplasma sequences and previous hemotrophic *Mycoplasma* sequences obtained from canids, felids, and wild animals from Chile, as previously discussed [28].

Even though hemoplasmas detected in rodents from Chile did not share many similarities with other hemoplasmas detected in mammals at a higher level, bat hemoplasmas from Chile represented a sister clade, which was supported by high posterior probability values (99). Hemoplasmas from bats may be a common ancestor for rodent hemoplasmas. Recently, bats were incriminated as the ancestral hosts of all mammal-related *Bartonella* [82] and appear to be responsible for the early geographic expansion and diversification of the genus. This could be the case for hemoplasmas and further investigation is required in this field.

## 5. Conclusions

This is the first report of hemoplasmas in synanthropic and wild rodents from Chile. A moderate occurrence for hemoplasmas and the presence of two remarkably related genotypes, similar to those previously detected in Brazil, were found in the present study. Hemoplasmas were more prevalent in wild rodents compared to synanthropic ones. The detected 16S rDNA hemoplasma sequences were specific for rodents and were not shared with those previously reported in other mammal species. This preliminary survey calls for more comprehensive studies on the epidemiology and genetics of hemoplasmas in rodents from Chile. Future studies should include a broader number of rodents from distinct regions in Chile, and evaluate hemoplasma DNA recombination and the relatedness of hemoplasmas found in bats and rodents.

## Figures and Tables

**Table 1:** Summarized information on the different primer sets, amplification cycles, and product size used in conventional PCR assays.

Primer	Target Gene	Sequence (5′-3′)	Amplification Cycles		Amplicon Size (bp)	Reference
IRBP-CF-FWD	*irbp*	TCCAACACCACCACTGAGATCTGGAC	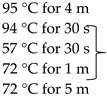	35 cycles	227	Ferreira et al., 2010
IRBP-CF-REV		GTGAGGAAGAAATCGGACTGGCC				
HemMyco16S-41s	16S rRNA	GYATGCMTAAYACATGCAAGTCGARCG	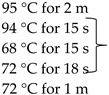	55 cycles	800	Maggi et al., 2013
HemMyco16S-938as		CTCCACCACTTGTTCAGGTCCCCGTC				

*irbp*: Interphotoreceptor retinoid-binding protein.

**Table 2:** Polymorphism and genetic diversity of 16S rDNA hemotrophic *Mycoplasma* spp. sequences detected in rodents from the Valdivia province, Chile (A), and combined with those previously detected in rodents from several worldwide regions (B).

	Species	(bp)	N	VS	GC %	G	Gd (mean ± SD)	π (mean ± SD)	K
A	*Mycoplasma* spp.	510	8	1	0.466	2	0.25 ± 0.180	0.0005 ± 0.0003	0.25
B	*Mycoplasma* spp.	876	36	112	0.462	18	0.94 ± 0.0006	0.068 ± 0.005	32.97

N; the number of analyzed sequences; VS, number of variable sites; GC, G+C content; g, number of genotypes; Gd, genotypes diversity; SD, standard deviation; π, nucleotide diversity (per site = PI); K, the average number of nucleotide difference.

## Supplementary Materials

The following are available online at https://www.mdpi.com/2076-2607/8/10/1493/s1, Table S1: Hemoplasma 16S rDNA sequences used in genotype and Splits network analyses.
