# Supplementary material for: Molecular Survey and Genetic Diversity of Hemoplasmas in Rodents from Chile
Source: Microorganisms. 2020 Sep 29;8(10):1493. doi: 10.3390/microorganisms8101493 (PMC7601721; doi:10.3390/microorganisms8101493)
Supplement: Supplementary file 1 [file microorganisms-08-01493-s001.pdf]

Appendix A

Supplemental Table 1. Hemoplasma 16S rDNA sequences used in genotype and Splits network analyses.

ACCESSION NUMBER	ORGANISM	HOST	COUNTRY
KT215622	Uncultured *Mycoplasma*	*Delomys dorsali*	Brazil
KT215623	Uncultured *Mycoplasma*	*Necromy lasiurus*	Brazil
KT215621	Uncultured *Mycoplasma*	*Akodon* sp	Brazil
KT215620	Uncultured *Mycoplasma*	*Akodon* sp	Brazil
KT215626	Uncultured *Mycoplasma*	*Rhipidomys macrurus*	Brazil
KT215629	Uncultured *Mycoplasma*	*Oligoryzomys nigripes*	Brazil
KT215643	Uncultured *Mycoplasma*	*Rattus rattus*	Brazil
KT215640	Uncultured *Mycoplasma*	*Rattus rattus*	Brazil
KT215636	Uncultured *Mycoplasma*	*Akodon* sp	Brazil
KT215637	Uncultured *Mycoplasma*	*Akodon* sp	Brazil
FJ667773	Uncultured *Mycoplasma*	*Hydrochoerus hydrocaeris*	Brazil
KC863983	Uncultured *Mycoplasma*	*Micromys minutus*	Hungary
FJ667774	Uncultured *Mycoplasma*	*Hydrochoerus hydrocaeris*	Brazil
KJ739312	Uncultured *Mycoplasma*	*Rattus norvegicus*	Hungary
AB752303	*Mycoplasma* sp.	*Rattus norvegicus*	Japan
KJ739311	Uncultured *Mycoplasma*	*Rattus norvegicus*	Hungary
AY171918	*Mycoplasma coccoides*	*Mus musculus*	UK
AB918692	C. M. haemomuris musculi	*Apodemus argenteus*	Japan
MN423261	Uncultured *Mycoplasma* sp	*Rattus norvegicus*	Brazil
MN423262	Uncultured *Mycoplasma* sp	*Rattus norvegicus*	Brazil
MN423263	Uncultured *Mycoplasma* sp	*Rattus rattus*	Brazil
MT345318*	Uncultured *Mycoplasma* sp	*Mus musculus*	Chile

MT345319*	Uncultured *Mycoplasma* sp	*Abrothrix olivaceus*	Chile
MT345320*	Uncultured *Mycoplasma* sp	*Abrothrix olivaceus*	Chile
MT345321*	Uncultured *Mycoplasma* sp	*Abrothrix olivaceus*	Chile
MT345322*	Uncultured *Mycoplasma* sp	*Abrothrix longipilis*	Chile
MT345323*	Uncultured *Mycoplasma* sp	*Abrothrix olivaceus*	Chile
MT345324*	Uncultured *Mycoplasma* sp	*Abrothrix olivaceus*	Chile
MT345325*	Uncultured *Mycoplasma* sp	*Abrothrix olivaceus*	Chile

*: present study sequences.
